# Amodal completion instead of predictive coding can explain activity suppression of early visual cortex during illusory shape perception

**DOI:** 10.1167/jov.21.5.13

**Published:** 2021-05-14

**Authors:** Chuyao Yan, Alexis Pérez-Bellido, Floris P. de Lange

**Affiliations:** 1 Donders Institute for Brain, Cognition, and Behaviour, Radboud University, Nijmegen, the Netherlands; 2 Department of Cognition, Development and Educational Psychology, University of Barcelona, Barcelona, Spain; 3 Institute of Neurosciences, University of Barcelona, Barcelona, Spain

**Keywords:** amodal completion, illusory shape perception, kanizsa illusion, neural adaptation, predictive coding

## Abstract

A set of recent neuroimaging studies observed that the perception of an illusory shape can elicit both positive and negative feedback modulations in different parts of the early visual cortex. When three Pac-Men shapes were aligned in such a way that they created an illusory triangle (i.e., the Kanizsa illusion), neural activity in early visual cortex was enhanced in those neurons that had receptive fields that overlapped with the illusory shape but suppressed in neurons whose receptive field overlapped with the Pac-Men inducers. These results were interpreted as congruent with the predictive coding framework, in which neurons in early visual cortex enhance or suppress their activity depending on whether the top-down predictions match the bottom-up sensory inputs. However, there are several plausible alternative explanations for the activity modulations. Here we tested a recent proposal (Moors, 2015) that the activity suppression in early visual cortex during illusory shape perception reflects neural adaptation to perceptually stable input. Namely, the inducers appear perceptually stable during the illusory shape condition (discs on which a triangle is superimposed), but not during the control condition (discs that change into Pac-Men). We examined this hypothesis by manipulating the perceptual stability of inducers. When the inducers could be perceptually interpreted as persistent circles, we replicated the up- and downregulation pattern shown in previous studies. However, when the inducers could not be perceived as persistent circles, we still observed enhanced activity in neurons representing the illusory shape but the suppression of activity in neurons representing the inducers was absent. Thus our results support the hypothesis that the activity suppression in neurons representing the inducers during the Kanizsa illusion is better explained by neural adaptation to perceptually stable input than by reduced prediction error.

## Introduction

**Figure 1.**
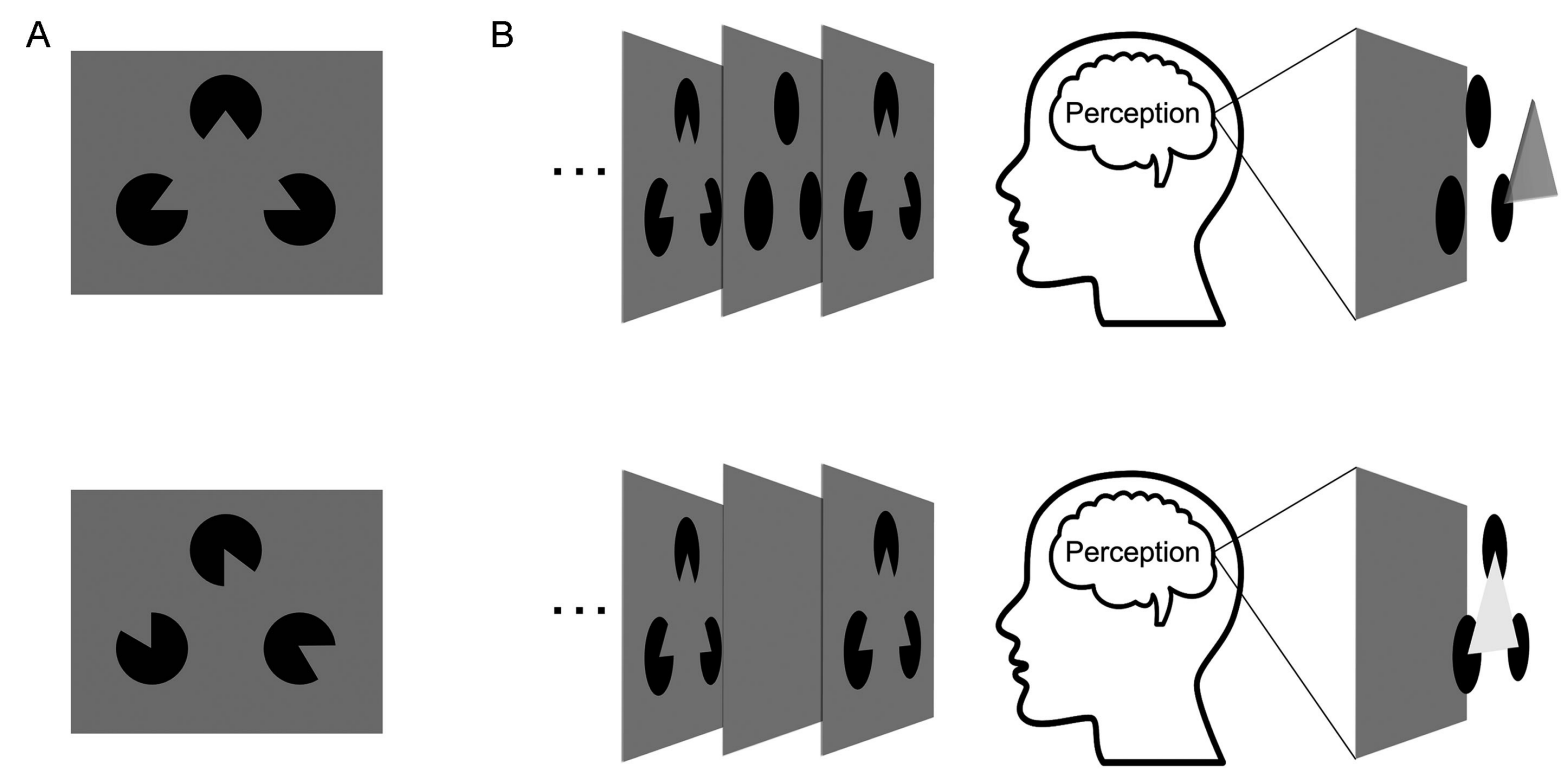
(A) Example of a “Kanizsa” stimulus (upper), in which three “Pac-Men” inducers were aligned such that an illusory triangle could be perceived, and a non-illusory control stimulus (lower) in which the configuration of the three inducers does not allow the perception of the illusory triangle. (B) In the circles condition (upper), the alternating sequence of the inducers and black circles leads to the perception that three circles are occluded by a triangle (dark gray), whereas in the no circles condition (lower), the inducers alternated with a blank screen, thus the three inducers should be uniquely perceived as the corners that give rise to an illusory triangle (light gray).

An essential function of the visual system is to organize the different visual features into coherent shapes, allowing us to perceive objects rather than the individual array of edges and lines that comprise the retinal input. Neurocomputational models such as predictive coding (Friston, 2005; Rao & Ballard, 1999) propose that the brain constantly generates predictions to explain its inputs, resulting in prediction errors, which are then used to update the predictions. This account can potentially explain why shape perception can sometimes lead to enhanced (Altmann et al., 2003; Meng et al., 2005; Muckli et al., 2005; Seghier & Vuilleumier, 2006) but in other cases reduced (Fang et al., 2008; He et al., 2012; Murray et al., 2002) neural activity in early visual areas, given that the effect of feedback signals is dependent on whether the signal is met by congruent bottom-up input (Kok & de Lange, 2014). To test this hypothesis, Kok and de Lange (2014) used functional magnetic resonance imaging (fMRI) to investigate how the brain represents an illusory visual percept when experiencing the “Kanizsa” illusion (Kanizsa, 1976). In this illusion, three Pac-Men, when properly aligned, form the corners that give rise to the perception of the boundaries of an illusory triangle (Figure 1A). They observed that compared with viewing control configurations such as rotated a version of the Pac-Men that did not give rise to the illusion, neurons whose receptive fields fell onto the illusory triangle increased their activities, whereas the neurons that processed the inducers decreased their activities during illusory shape perception. These results were speculatively interpreted within a predictive coding framework, as the increased activity can be explained by an increase in both prediction units (a triangle is expected) and prediction error units (there is no bottom-up input consistent with the prediction). The decreased activity was hypothesized to be because of the match between top-down predictions and bottom-up input at the inducer locations, leading to a reduced prediction error.

Recently, it has been put forward that the suppression at the inducers could be explained by an alternative plausible mechanism: neural adaptation to perceptually stable input (Moors, 2015). Because of the specific trial sequence used in the Kok and de Lange (2014) experiment (see also Lee & Nguyen, 2001), in the Kanizsa condition the participants could perceive the inducers as persistent circles intermittently occluded by an illusory triangle. This perceptual interpretation would not hold for the control condition, in which the inducers would be perceived uniquely as Pac-Men. Therefore during occlusion periods, participants could amodally complete the inducers as circles in the illusory Kanizsa condition but not in the control condition. As such, amodally completed circles could generate stronger neural adaptation in the illusory compared with the non-illusory condition, thus potentially explaining the suppression effects at the inducer locations.

**Figure 2.**
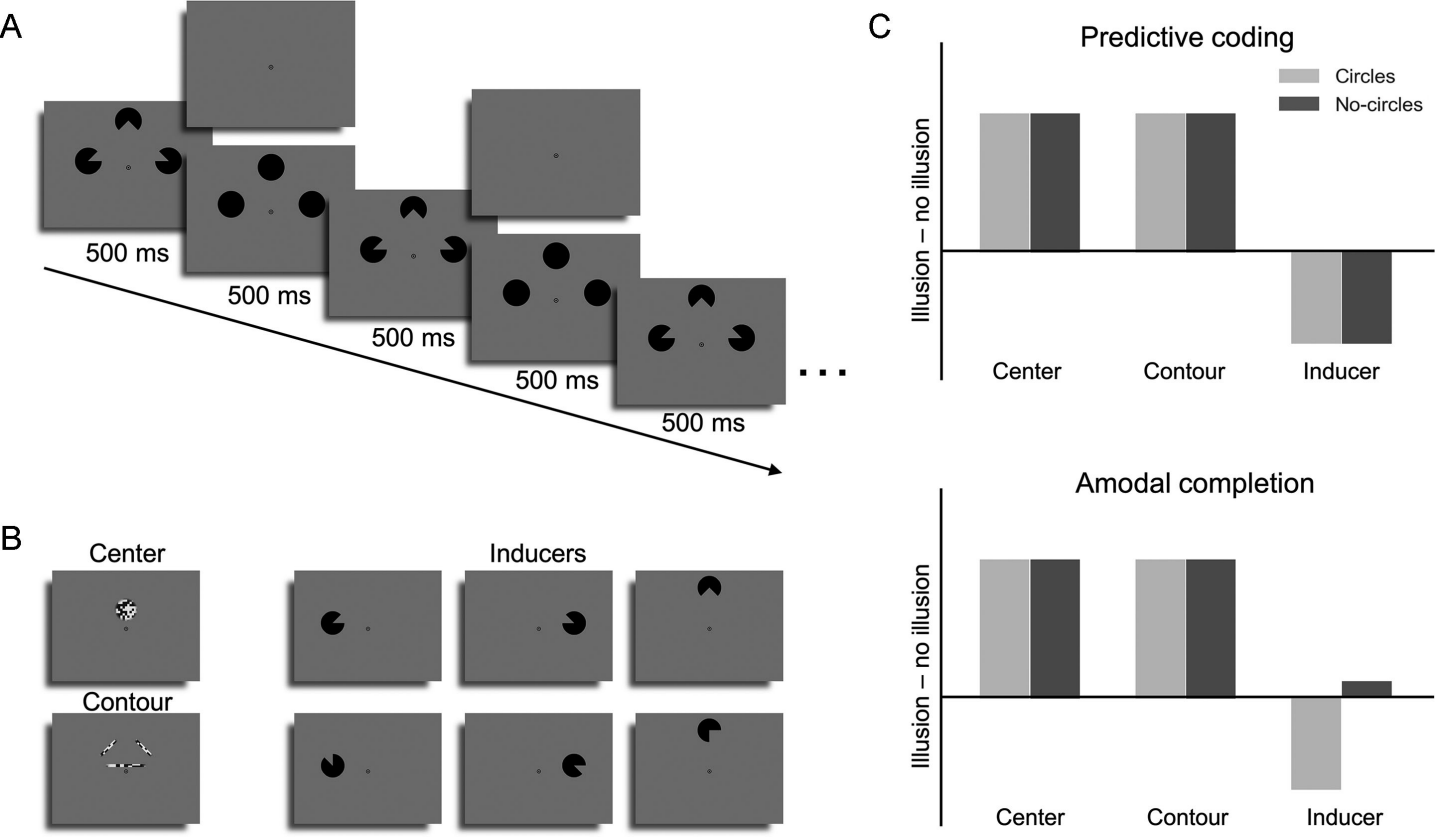
(A) An example of Kanizsa trial with Pac-Men inducers alternating with or without black circles (circles and no circles condition). (B) Functional localizers. Two checkerboard textures (left) were used to select those voxels that respond to the illusory triangle (upper) and its contours (lower), respectively. Six Pac-Men (right) were used to select those voxels that respond to the inducers. The three upper inducers conform the illusory triangle condition, whereas the lower inducers conform the non-illusory control condition. (C) Predictions for the two hypotheses. According to predictive coding (upper), there should be enhancement in the locations related to illusory triangle and the suppression in the locations related to inducers despite the presentation of circles. By the contrast, according to amodal completion explanation (lower), the suppression is produced by an adaptation effect to the amodally completed circles during the alternating sequence. Therefore the suppression should appear in circles condition but disappear in no circles condition.

In the present study, we examined this alternative explanation using a slight modification of the paradigm used by Kok and de Lange (2014) by including an additional experimental manipulation: during the experiment, the inducers could either alternate with full black circles or a blank screen containing no circles. Therefore in the inducers alternating with the black circles condition (circles condition), the inducers can be perceived as persistently present circles (Figure 1B, upper panel). However, in the inducers alternating with a blank screen condition (no circles condition), the circles should not be perceived as persistently present, preventing amodal completion to arise (Figure 1B, bottom panel). We reasoned that this manipulation allowed us to test whether the suppressive effects related to the inducers reflect reduced prediction error or neural adaptation to perceptually stable input. Specifically, we predicted that if the suppressive effects reported for the inducers in previous Kanizsa studies (Kok et al., 2016; Kok & de Lange, 2014; Utzerath et al., 2019) correspond to top-down fulfilled predictions, the pattern of results should not qualitatively change as a function of the presence or absence of the circles (Figure 2C, upper panel). By contrast, if the suppressive effects taking place at the inducers are generated by neural adaptation of amodally completed circles, the suppression should disappear when the inducers are not perceived as persistently present circles, that is, during the no circles condition (Figure 2C, bottom panel). We expected neural enhancement at the center of the illusory triangle in both conditions.

## Methods

### Participants

Seventeen healthy human subjects (11 women, age 22.4 ± 3.1 years) from the Radboud Research Participation System gave written informed consent to participate in this study. All participants were prescreened for MRI compatibility and had normal or corrected-to-normal vision. This study was approved by the local ethics committee (CMO Arnhem-Nijmegen, Radboud University Medical Center) under the general ethics approval. Participants were compensated with 20 euros for study participation. One subject left the experiment because of excessive tiredness (total sample size n = 16).

### Stimuli

Stimuli were generated using MATLAB R2017b (The MathWorks, Natick, MA; RRID:SCR_001622) in combination with PsychToolbox (Brainard, 1997) During experiment, the stimuli were presented on a rear projection screen (LC-XL 100 beamer, EIKI, Osaka, Japan, with a resolution of 1024 × 768 pixels and a refresh rate of 60 Hz), visible using an adjustable mirror. The configurations consisted of three black circles with missing wedges (Pac-Men inducers; 4° diameter) presented on a mid-gray background. The upper inducer (90° wedge cutout) was displayed 6.5° above a fixation bull's-eye (0.7° diameter) that was centered on the screen. The left and right inducers (45° wedge cutout) were arranged horizontally 12° apart and displayed at 0.5° above the fixation. During the main task, these inducers were rotated to form two types of inducer configurations. In the Kanizsa configuration (Figure 1A), three inward-facing inducers were aligned such that they induced the perception of an illusory triangle. In control configurations, the inducers were rotated such that non-illusory figure could be perceived while keeping the overall configuration similar to the illusory condition.

### Experimental design

All participants completed the main task and a functional localizer in the MRI scanner. In the main task, we used a blocked design in which the stimuli were presented in approximately 14-second duration blocks. Each block consisted of the repetition of one of the four possible Pac-Men configurations at a rate of 1 Hz (500 ms on, 500 ms off; see Figure 2A). In half of the blocks, the participants were presented with Kanizsa configurations (illusory condition), whereas in the other half with control configurations (non-illusory condition). Moreover, and orthogonally to the inducers configuration, in half of the blocks the three inducers alternated with black circles (circles condition), and in the other half the inducers alternated with an empty background (no circles condition, Figure 2A). In total, there were four conditions conforming a 2 × 2 factorial design. The different condition blocks were presented in a pseudorandomized order ensuring that each configuration was presented once before the four conditions would be repeated again. Between stimulation blocks, there were approximately 10-second fixation blocks in which only the bull's-eye was displayed on the screen. This fixation period established a baseline response in the brain, against which the figure conditions could be contrasted.

During the task, the fixation bull's-eye dimmed randomly in brightness 1.5 times per trial on average. To draw their attention away from the illusory shapes, participants were required to fixate at the bull's-eye and respond by button press whenever they detected a brightness change. Subjects performed four runs (∼10 min per run). In total, participants completed 192 blocks, of which 24 corresponded to each Pac-Men configuration condition and 96 to the fixation condition. To ensure that participants keep fixation and pay attention to the task, an infrared eye tracker (SensoMotoric Instruments, Teltow, Germany) was used to monitor eye position.

### Functional localizer

In a block design, we used two types of functional localizers to identify those voxels whose receptive fields spatially overlapped with the illusory triangle and inducer stimuli, respectively. To localize voxels responsive to the illusory triangle center (Kok & de Lange, 2014) and contours (Mendola et al., 1999; Stanley & Rubin, 2003), we used random changing checkerboard textures (Figure 2B, left panel). The center localizer (4° diameter) was a checkerboard circle presented on 2.5° above fixation, whereas the contour localizer consisted of three checkerboard bars (left and right bars: 0.5° × 4°; lower bar: 0.5° × 7°). To localize those voxels sensitive to the inducer stimuli, we used six black inducers (Figure 2B, right panel) akin to the ones conforming the illusory and control configurations. There were also fixation blocks, during which only the fixation bull's-eye was displayed for approximately 10 seconds.

During each functional localizer block, the stimuli were presented at 2 Hz for approximately 14 seconds. The localizer blocks were presented one after the other in a pseudorandomized order, with fixation blocks after each eighth localizer block. In total, participants performed three runs (∼10 min per run) with 108 localizer blocks, of which 12 blocks corresponded to each localizer stimulus and 12 blocks to the fixation periods. Participants performed the same detection task as in the main task.

### fMRI data acquisition

Functional and anatomic images were collected on a 3T Skyra MRI system (Siemens, Munich, Germany), using a 32-channel head-coil. Functional images were acquired using a T2*-weighted multiband-3 sequence to acquire partial brain volumes aligned to maximize coverage of early visual areas (TR/TE = 825/32 ms, 27 slices, voxel size = 2 mm isotropic, 55° flip angle, A/P phase encoding direction). Anatomic images were acquired with a T1-weighted MP-RAGE (GRAPPA acceleration factor = 2, TR/TE = 2300/3.03 ms, voxel size 1 mm isotropic, 8° flip angle).

### fMRI data preprocessing

fMRI data preprocessing was performed using FSL 5.0.9 (FMRIB Software Library, Oxford, UK; RRID:SCR_002823). The preprocessing pipeline included brain extraction (BET), motion correction (MCFLIRT), and temporal high-pass filtering (128 s). The spatial smoothing was not performed because we need voxel-by-voxel resolution for the adjacent localizers. Functional images were registered to the structural image using boundary-based registration (BBR) as implemented in FLIRT. For each run, the first 10 volumes of each run were discarded to allow for signal stabilization.

### fMRI data analyses

To identify neural modulations associated with the illusory triangle and inducers visual processing we modeled the functional data using a general linear model (GLM) performed in FSL FEAT. For the main task, we modeled separate regressors for illusory and non-illusory figures within circles and no circles condition, and the fixation condition. For the localizer, we modeled the eight stimuli localizers (Figure 2B) and fixation condition. All trials were modeled with corresponding duration of stimuli presentation (14 s for the stimuli trials, 10 s for the fixation trials). In addition, nuisance regressors were added for both main task and localizer, including first-order temporal derivatives for all modeled event types, and 24 motion regressors (six motion parameters, the derivatives of these motion parameters, the squares of the motion parameters, and the squares of the derivatives; comprising the FSL standard + extended set of motion parameters).

### Definition of region of interest

V1 and V2 were defined based on each participant's individual anatomic image. Freesurfer 6.0 (General Hospital Corporation, Boston MA; RRID:SCR_001847) was used to extract labels (left and right) per subject based on their anatomic image, which were transformed into native space using ‘mri_label2vol’ and merged into a bilateral mask. Subsequently, we used Z-statistics maps obtained from the functional localizer to select those voxels that were responsive to the three locations (i.e., illusory triangle, its contours and the inducers) against the fixation condition. The checkerboard localizers were more effective in stimulating the visual cortex and induced larger activity than the flashed Pac-Men stimuli. To balance the large univariate activity differences between localizers, we normalized the Z-statistics maps using a z-score transformation before generating the contrast maps for each location (i.e., inducers over illusory triangle and its contours). One hundred voxels were selected as defined by the highest Z-statistics in the respective contrast map. This process yielded three regions of interest (ROIs) in each of V1 and V2. To verify that our results were not unique to the specific ROI size, we repeated all ROI analyses with ROI masks ranging from 50 to 200 voxels in steps of 50 voxels.

### ROI analyses

ROI-based analyses were conducted in native space. The parameter estimates of the fixation condition were subtracted from the other conditions of interest to generate contrast maps. Subsequently the contrasted parameter estimates within each ROI and condition were extracted and used to calculate the mean parameter estimate over the selected voxels. The averaged parameter estimates within each ROI were transformed to percentage of signal change for subsequent statistical analyses.

First, we tested for the existence of an up- and downregulation of neural activity during illusion perception. The data were submitted to a 2 × 3 repeated measures analysis of variance (RM ANOVA) with inducers configurations (illusory figure and non-illusory figure) and location (center, contour, and inducer) as factors for the circles and no circles conditions in V1 and V2 anatomic regions, respectively. Second, we directly contrasted whether the strength of the neural responses induced by the illusory triangle depended on interpretation of the inducers as stable circles or corners of the triangle. To do that we indexed the illusion strength by calculating the difference in parameter estimates between the illusory and non-illusory conditions for each location. Positive values of the difference indicated an enhanced neural activity, whereas negative values indicated a suppression. These values were compared between locations split into circles and no circles conditions. Thus a 2 × 3 RM ANOVA with display type (circles and no circles) and location (center, contour, and inducer) as factors was used for analysis. Main effects across conditions were calculated for the neural activity within each location using two-sided paired *t*-tests. As applicable, partial eta-squared (*η*^2^) and Cohen's *d* were calculated as measures of effect size for the ANOVA and *t*-tests, respectively. Furthermore, Bayesian analysis was used to evaluate any nonsignificant tests. All statistical testing was performed using Pingouin 0.2.9 (Vallat, 2018) in Python 3.7.4 (Python Software Foundation; RRID:SCR_008394).

### Software

Stimuli were presented using PsychToolbox (Brainard, 1997) running on MATLAB R2017b (The MathWorks; RRID:SCR_001622). MRI data preprocessing and analysis was performed using FSL 5.0.9 (FMRIB Software Library; www.fmrib.ox.ac.uk/fsl; RRID:SCR_002823) and Freesurfer 6.0 (General Hospital Corporation; RRID:SCR_001847). Python 3.7.4 (Python Software Foundation; RRID:SCR_008394) was used for data processing, visualization, and statistical tests with the following libraries: NumPy 1.17.2 (van der Walt et al., 2011), Pandas 0.25.1 (McKinney, 2010), Nibabel 2.5.1 (Brett et al., 2019), Matplotlib 3.1.1 (Hunter, 2007), and Pingouin 0.2.9 (Vallat, 2018).

## Results

### Behavioral performance

Participants showed near ceiling-level performance in detecting the fixation dimming events in all the conditions (mean hit rate = 95.6% ± 6.7%, mean ± standard deviation [SD]). Moreover, their performance was similar for illusion and no illusion trials in both the circles (hit rate: 96.0% ± 5.4% vs. 94.9% ± 8.2%; reaction time [RT]: 482 ± 90 vs. 483 ± 94 ms, mean ± SD) and the no circles condition (hit rate: 96.0% ± 6.5% vs. 96.0% ± 6.8%; RT: 481 ± 87 vs. 482 ± 92 ms, mean ± SD).

### Up- and downregulation of neural activity in the circles condition

We first examined whether the circles condition, which reproduces the same experimental paradigm used in previous studies (Kok & de Lange, 2014; Utzerath et al., 2019), replicates their up- and downregulation pattern of neural activity (i.e., enhanced activity in those voxels whose receptive fields overlap with the illusory triangle, and suppressed activity in those voxels whose receptive fields overlap with the inducers of the illusory triangle). Two RM ANOVAs were performed to inspect how the average BOLD signal activity within each visual region (V1 and V2) changed as a function of the location (center, contour, and inducers) and inducer configuration (illusory figure and non-illusory figure). The significant main effects of location (V1: *F*_(2,30)_ = 45.675, *p* < 0.001, *η*^2^ = 0.753; V2: *F*_(2,30)_ = 4.407, *p* = 0.018, *η*^2^ = 0.236) revealed that the magnitude of the BOLD signal responses differed between locations. Indeed, post hoc *t*-tests showed significantly larger BOLD responses at the inducers location compared with the Kanizsa figure (center and contour locations) in both V1 and V2 (all *p* < 0.01). This is entirely expected, as there was bottom-up input only at the inducer locations. We also observed significant interactions between location and inducer configuration (V1: *F*_(2,30)_ = 20.790, *p* < 0.001, *η*^2^ = 0.581; V2: *F*_(2,30)_ = 60.458, *p* < 0.001, *η*^2^ = 0.801), suggesting that the illusion modulated activity differently in the three locations. To unpack this interaction, we used multiple paired *t*-tests to compare the BOLD signal in the illusory versus control condition for each location and visual region. The results indicated that the illusory condition upregulated the neural activity of those voxels responsive to the illusory shape at the center location (V1: *t*_(15)_ = 1.424, *p* = 0.175, *d* = 0.130; V2: *t*_(15)_ = 5.256, *p* < 0.001, *d* = 0.345) and the contour location (V1: *t*_(15)_ = 4.821, *p* < 0.001, *d* = 0.447; V2: *t*_(15)_ = 6.767, *p* < 0.001, *d* = 0.572), but downregulated the activity at the inducers location (V1: *t*_(15)_ = −2.993, *p* = 0.009, *d* = −0.158; V2: *t*_(15)_ = −5.149, *p* < 0.001, *d* = −0.218). All results were robustly present, independent of the amount of voxels included in the ROIs (with the exception of the enhancement at center location in V1, which only reached statistical significance with an ROI size of 50 voxels, see Figure 4).

In an exploratory analysis, we compared the magnitude of the illusion-induced activity modulation between the center and contour locations in the circles condition. To quantify the magnitude of the illusion-induced activity modulation, we subtracted the BOLD activity in the non-illusory from the illusory condition (see Figure 5A). Paired *t*-tests revealed that the voxels elicited larger enhancements in the contour location than the center location (V1: *t*_(15)_ = 3.192, *p* = 0.006, *d* = 0.917; V2: *t*_(15)_ = 4.781, *p* < 0.001, *d* = 1.146), suggesting that the neurons in the early visual cortex are more sensitive to illusory contours than to the illusory surface (Lamme, 1995; Peterhans & Heydt, 1989). In addition, we also tested whether the magnitude of the illusion-induced activity modulation differed across visual regions. Our results showed that in general, the activity enhancement during the illusory shape at both contour and center location was stronger in V2 than V1 (center: *t*_(15)_ = 4.508, *p* < 0.001, *d* = 0.988; contour: *t*_(15)_ = 7.011, *p* < 0.001, *d* = 1.157, see Figure 5A), whereas there was no significant difference in terms of the suppressive effects to the inducers (*t*_(15)_ = −1.918, *p* = 0.074, *d* = −0.383). These results are compatible with previous findings demonstrating that V2 showed stronger effects to the Kanizsa illusion than V1 (Anzai et al., 2007; Heydt et al., 1984).

### Up- and downregulation of neural activity in the no circles condition

**Figure 3.**
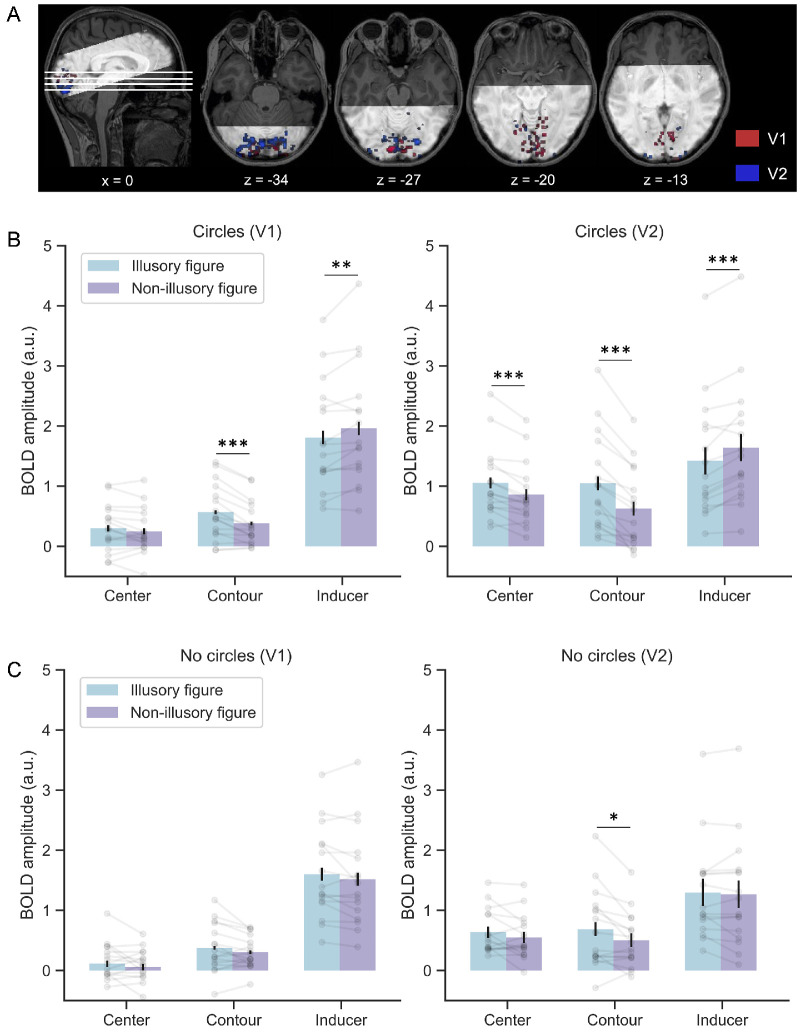
Illusory shape modulated activity across locations in early visual cortex (V1 and V2). (A) Represents the 300 most responsive voxels to the three functional localizers in V1 (red) and V2 (blue) from a representative participant. (B) Represents the average parameter estimates ± standard error in each ROI (100 voxels) for response to illusory (cyan) and non-illusory (purple) figures across all locations for circles condition. Gray dots and lines indicate individual observations. Error bars indicate standard error. Significance levels correspond to *p* < 0.05 (*), *p* < 0.01 (**), and *p* < 0.001 (***). (C) Represents average parameters estimates ± standard error for the no circles condition. Same labels as in (B).

**Figure 4.**
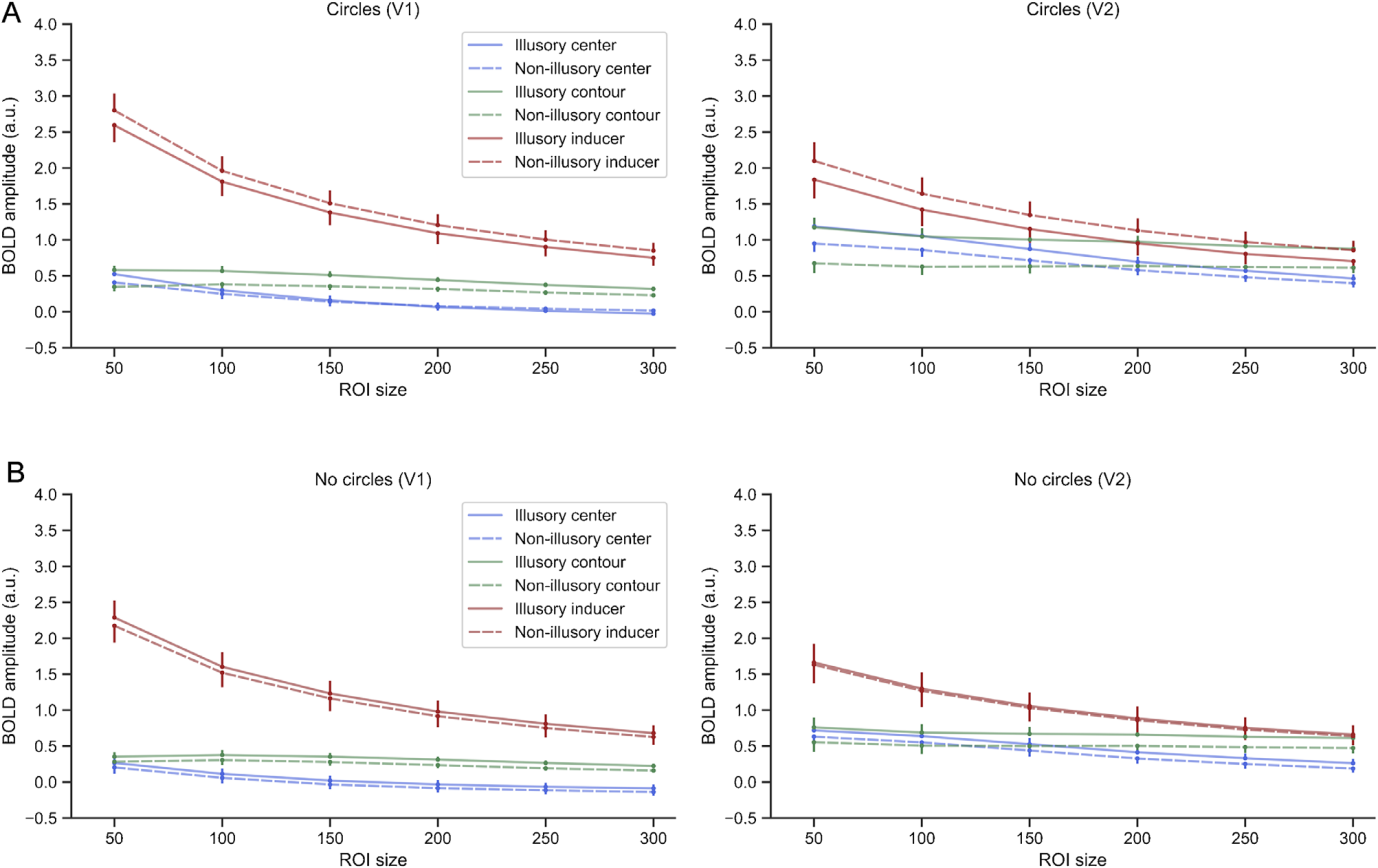
Illusory shape modulated activity across different ROI sizes in early visual cortex (V1 and V2). (A) Represents the average parameter estimates ± standard error of all locations for response to illusory (solid lines) and non-illusory (dashed lines) figures across all ROI sizes for circles condition. Error bars indicate standard error. (B) Represents average parameters estimates ± standard error for the no circles condition. Same labels as in (A).

Next, following the same analysis pipeline we evaluated whether the enhancement and suppression pattern reported in the circles condition replicates when the inducers are not interpreted as persistently present circles. Again, we observed stronger neural activity at the inducers compared with the center and contour locations, confirmed by the main effect of location (V1: *F*_(2,30)_ = 43.945, *p* < 0.001, *η*^2^ = 0.746; V2: *F*_(2,30)_ = 6.802, *p* = 0.004, *η*^2^ = 0.312). However, the differential up- and downregulation of neural activity between regions was present only in V2 (*F*_(2,30)_ = 9.740, *p* < 0.001, *η*^2^ = 0.394), but not significant in V1 (*F*_(2,30)_ = 0.332, *p* = 0.720, *η*^2^ = 0.022). This result is consistent with the exploratory analyses described earlier and previous studies (Anzai et al., 2007; Heydt et al., 1984) in showing a stronger involvement of V2 compared with V1 during the processing of illusory shapes. Paired *t*-tests in V2 revealed that the enhancement was present at the contour location (*t*_(15)_ = 2.639, *p* = 0.019, *d* = 0.329) but not significant at the center location (*t*_(15)_ = 1.680, *p* = 0.114, *d* = 0.236). Importantly, no significant suppression was observed at the inducer location (*t*_(15)_ = 0.633, *p* = 0.536, *d* = 0.033). Indeed, a Bayesian statistical analysis yielded anecdotal support (BF_10_ = 0.810) and moderate support (BF_10_ = 0.305) for an absence of illusory effect at the center and inducer location, respectively. Thus our analyses of the no circles condition show a significant upregulation pattern in V2, primarily driven by a BOLD signal enhancement at the contour location during Kanizsa trials. A similar trend was observed in the center location in V2 and the contour and center locations in V1 (Figures 3B and 4). However neither in V1 or V2 the inducers showed a suppression pattern. These results suggest that when the inducers are not perceived as stable circles, the suppression effects previously reported at the inducers in the circles condition were no longer present.

### Amodal completion explains activity suppression of early visual cortex during illusory shape perception

We explored how the presentation of circles in-between Kanizsa configurations (circles and no circles condition) modulates the neural response evoked by the illusory triangle at early visual regions. For each visual region (V1 and V2) and ROI condition (center, contour, and inducer) we performed an RM ANOVA to test whether the average BOLD signal activity changed as a function of the display type (circles and no circles) and inducer configuration (illusory figure and non-illusory figure). In both V1 and V2, the analyses revealed that the magnitude of the BOLD signal responses were on average significantly higher in the circles compared with the no circles condition (all *p* < 0.01). We also observed significant interactions between display type and inducer configuration at the contour (V1: *F*_(1,15)_ = 9.378, *p* = 0.007, *η*^2^ = 0.394; V2: *F*_(1,15)_ = 14.106, *p* = 0.002, *η*^2^ = 0.485) and inducer ROIs (V1: *F*_(1,15)_ = 12.280, *p* = 0.003, *η*^2^ = 0.450; V2: *F*_(1,15)_ = 14.493, *p* = 0.002, *η*^2^ = 0.491) revealing differences in the strength of illusion between the circles and no circles conditions. However, no significant interaction was observed at the center (V1: *F*_(1,15)_ = 0.004, *p* = 0.951, *η*^2^ < 0.001; V2: *F*_(1,15)_ = 3.376, *p* = 0.09, *η*^2^ = 0.184). This may be owing to weaker effect of the illusion at the center compared with the contour ROI that might shadow any existing interaction.

**Figure 5.**
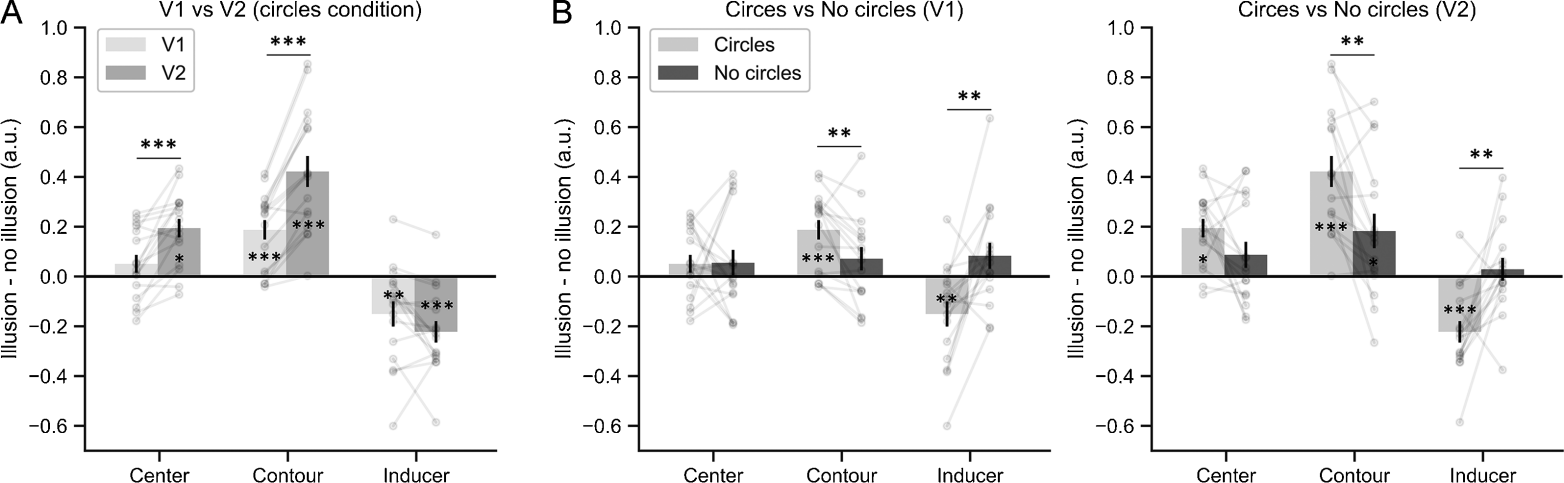
(A) Neural responses to illusion split into V1 (light livid) and V2 (dark livid) for all locations in the circles condition. (B) Neural responses to illusion (illusion minus no illusion) split into circles (light gray) and no circles condition (dark gray) for all locations in V1 (left) and V2 (right). Error bars indicate standard error. Significance levels correspond to *p* < 0.05 (*), *p* < 0.01 (**), and *p* < 0.001 (***).

To directly test our experimental hypotheses (Figure 2C), we compared the magnitude of the illusion-induced activity in the circles and no circles conditions across all locations by subtracting the BOLD activity in the non-illusory from the illusory condition (Figure 5B). RM ANOVAs were performed using location (center, contour, and inducers) and display type (circles and no circles) as factors. In line with predictions derived from the amodal completion hypothesis, we found significant interactions between location and display type in V1 and V2 (V1: *F*_(2,30)_ = 14.029, *p* < 0.001, *η*^2^ = 0.783; V2: *F*_(2,30)_ = 35.019, *p* < 0.001, *η*^2^ = 0.700). This result indicated that the neural effects induced by the illusion were modulated by the display types. Paired *t*-tests revealed that the enhanced neural activity to the illusion at contours was significantly larger in the circles than in the no circles condition (V1: *t*_(15)_ = 3.121, *p* = 0.007, *d* = 0.678; V2: *t*_(15)_ = 3.756, *p* = 0.002, *d* = 0.909), suggesting stronger illusory effects to the Kanizsa triangle when the inducers were alternated with circles. However, no significant difference was observed at the center location (V1: *t*_(15)_ = −0.062, *p* = 0.951, *d* = −0.024; V2: *t*_(15)_ = 1.837, *p* = 0.086, *d* = 0.602). This is consistent with our previous results that the effects were more robust at the illusory contours than the center. At the inducer locations, the suppressive effects were significantly weaker in the no circles condition compared with the circles condition (V1: *t*_(15)_ = −3.504, *p* = 0.003, *d* = −1.141; V2: *t*_(15)_ = −3.807, *p* = 0.002, *d* = −1.415). In line with previous analyses showing that suppressive effects were absent in the no circles condition, our results are better accounted by the amodal completion hypothesis.

**Figure 6.**
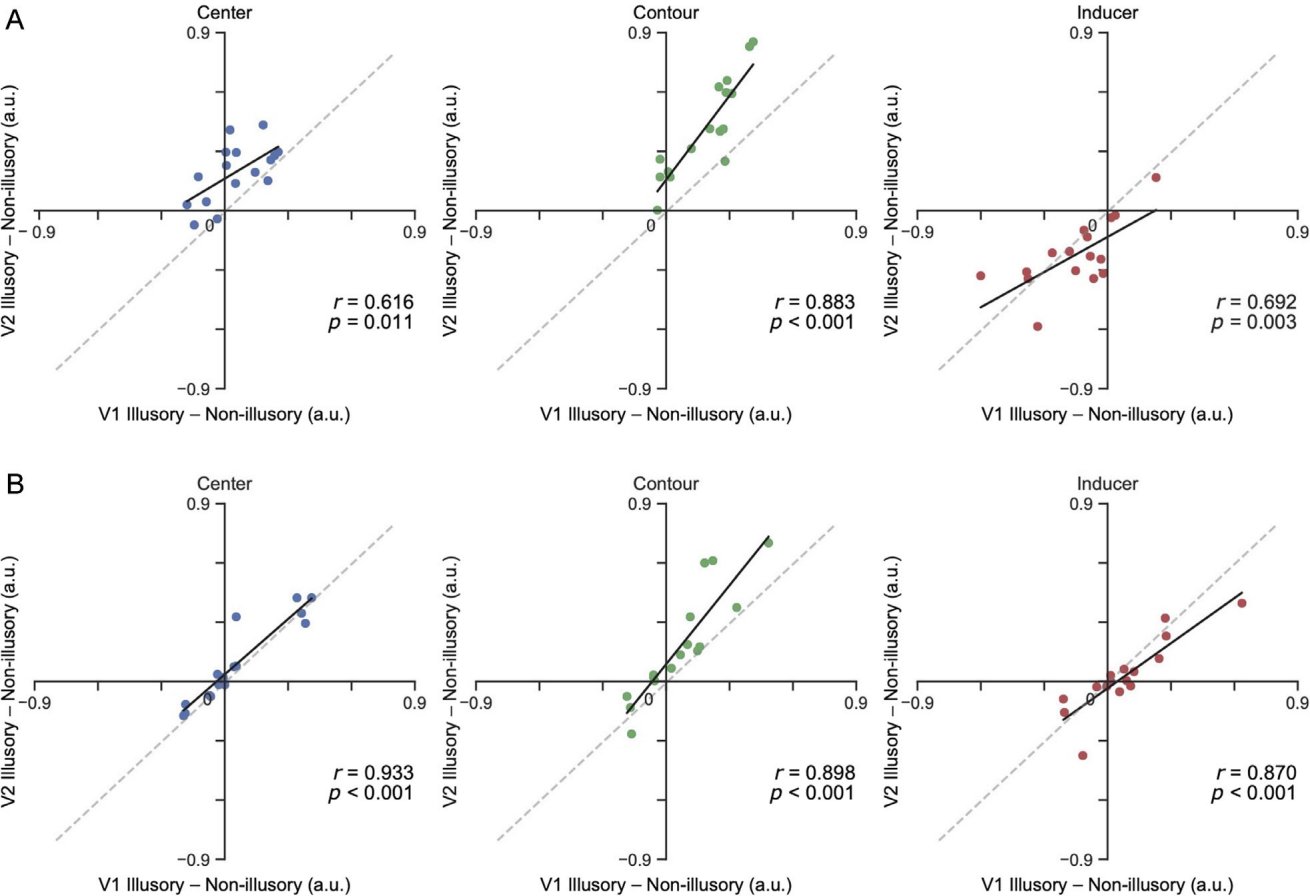
We found significant positive correlations between the neural effects induced by the Kanizsa illusion (illusory minus non-illusory condition) within V1 and V2 at all ROI locations. Most of the dots in the center and contour ROIs fall above the unity line, manifesting that the illusory neural effect tends to increase in V2 relative to V1. The black lines represent the correlation slopes for all functional localizers in the circles (A) and no circles (B) conditions. Colored dots indicate individual participants’ observations in V1 and V2 for each condition (blue, center; green, contour; and red, inducers). Statistics reflect Pearson correlations.

In addition, we correlated the size of the neural effects (illusory minus non-illusory condition) in V1 and V2 for each ROI to investigate whether the neural expression of the illusion was associated across visual regions. The results showed a strong correspondence in the direction of the effects between both visual regions in all the ROIs (all r > 0.616, *p* < 0.05) but manifested a general increment of the magnitude of the effects in V2 compared with V1 in the ROIs overlapping with the illusory triangle (Figure 6).

## Discussion

In the present study, we used fMRI to examine whether predictive coding or amodal completion better accounts for the activity suppression in early visual cortex during Kanizsa illusion perception. To dissociate between these two hypotheses, we manipulated the interpretation of the inducer circles as either being persistently present during the display of the illusion (circles condition) or not (no circles condition). In the circles condition, we replicated earlier findings (Kok & de Lange, 2014; Utzerath et al., 2019), that is, enhanced early visual activity in neurons that have their receptive field overlapping with the illusory shape, and suppressed activity in the neurons that had receptive fields overlapping with the inducers during illusion perception. However, when we modified the paradigm such that the inducers could not be perceived as persistently present circles (no circles condition), we still found enhanced activity at the illusory shape location (albeit of reduced magnitude), but the suppressive effects at the inducer locations were absent. Thus our results support the hypothesis that the suppressive effects at the inducer locations are reflecting neural adaptation to amodally completed circles, rather than a reduction in prediction error.

### Stronger illusory responses in circles than in no circles condition

We observed that in the absence of bottom-up input, both the center and contour regions of the illusory shape showed enhanced responses when the inducers were aligned such that they produced an illusory shape. This neural modulation constitutes a compelling example of feedback activation of early visual areas, and within V1 this feedback modulation has been specifically localized to the deep layers (Kok et al., 2016). Interestingly, our results showed a more robust enhancement of activity for the center and contour locations in the circles than in the no circles condition. The difference between these two conditions could be related to the perceptual interpretation of the inducers. In the circles condition, the inducers may be more easily perceived as three static “background” circles. Thus the participants might perceive the illusory triangle as a salient and segregated entity flickering on top of them. By contrast, in the no circles condition the inducers could be perceived as another visual event competing in salience with the illusory triangle during their presentation. Thus the saliency of the illusory shape might be diluted by the inducers, leading to a general reduction of the illusion strength. We cannot provide empirical evidence demonstrating that the perceptual strength of the illusion is weaker in the no circles compared with the circles condition. However, at phenomenologic level it seems that the illusion is stronger when the three inducers can be interpreted as part of a static background (see Supplementary Materials S1, S2, S3 and S4 for an animated demo of the different stimuli). It could be argued that the absence of activity suppression at the inducer locations may be caused by the overall weaker illusory effects in the no circles condition. Our results are not compatible with this explanation. As shown in Figure 3B, there was a significant enhancement in the contour region of V2 also during the no circles condition, probing the existence of measurable top-down neural modulations. In addition, the suppressive effects at the inducers were robustly absent (Figure 4) and in fact the pattern was reversed compared with the circles condition, rendering unlikely that any kind of inducers suppression was present in a reduced form.

We also found that the BOLD signal enhancement was more robust at the contour than at the center of the illusory triangle. This is likely because of the fact that most of the neurons in the early visual cortex are more sensitive to illusory contours than to the illusory surface (Lamme, 1995; Peterhans & Heydt, 1989). The generation of the illusion could proceed by an extension of the real edges of the inducers (Seghier & Vuilleumier, 2006) and a filling-in process that renders the surface of illusory figure for shape completion (Grossberg & Mingolla, 1985).

Finally, our results showed that the BOLD response in the circles condition was on average significantly larger than in the no circles condition. This may be because of the fact that, in the circles condition, the inducers remain on the screen during the entire trial, resulting in a sustained bottom-up drive compared with the no circles condition. Of note, the fact that there is no stronger (but in fact weaker) bottom-up driven BOLD modulation in the no circles condition, ensures that the lack of modulation in the no circles condition cannot be the result of a masking of the more subtle top-down related modulations owing to stronger bottom-up input. Thus the lack of top-down modulation in the no circles condition is not likely explained by the differences in bottom-up drive.

### Stronger illusory responses in V2 than in V1

Single-unit studies suggest that both V1 and V2 represent illusory contours (Grosof et al., 1993; Sheth et al., 1996; Sugita, 1999), although signals in V2 present more robust responses than in V1 (Anzai et al., 2007; Heydt et al., 1984). Importantly, the illusory contour response in V2 precedes the response in V1 (Lee, 2001), indicating that contour completion in V1 might arise from a feedback modulation from V2. Previous fMRI studies (Mendola et al., 1999; Stanley & Rubin, 2003) show that both early visual cortices (V1/V2) and higher-order lateral occipital cortex (LOC) are involved in Kanizsa figure processing, with a stronger activation to the illusion in LOC (Maertens et al., 2008; Mendola et al., 1999). Furthermore, electroencephalography and magnetoencephalography studies (Anken et al., 2018; Halgren et al., 2003; Knebel & Murray, 2012; Murray et al., 2004; Sugawara & Morotomi, 1991) suggest that the illusory effects within early visual cortex are the result of feedback from higher-order cortical areas. These findings are compatible with our own results (Figure 6) that showed positive correlations between the enhancement and suppressive effects in both visual regions across ROIs but larger illusion-dependent modulations in V2 compared with V1, implying a stronger feedback modulation in V2 (Anzai et al., 2007; Heydt et al., 1984). Unfortunately, we could not validate the involvement of LOC in the illusion generation as we used a scanning sequence that did not include coverage of that brain region.

### Amodally completed stimuli induce sensory adaptation

In the current study we have shown that the suppression by the Kanizsa illusion is caused by adaptation to the amodally completed stimuli, when interpreting the inducer stimuli as perceptually persistent circles. It has been found that neural adaptation can occur not only for physically presented stimuli (Kourtzi & Kanwisher, 2001; Tootell et al., 1998), but also for illusory contours (Montaser-Kouhsari et al., 2007). Our study extends these findings by showing that adaptation can also occur for amodally completed shapes. It is worth noting that in our experiment the amodal completion of circles was simultaneously accompanied by modal completion of the illusory triangle. Therefore it is difficult to disentangle the specific contribution of amodal completion adaptation and the modal completion of the illusory triangle to the observed neural modulations. Further experiments excluding the influence from modal completion will be needed to better understand how neural adaptation to the amodally completed stimuli unfolds in isolation.

## Conclusions

Recent neuroimaging studies observed that the perception of the Kanizsa illusion elicits both enhanced and suppressed neural modulations in different parts of the early visual cortex (Kok & de Lange, 2014). Suppression effects at the receptive fields overlapping with the inducers of the illusory triangle were interpreted as congruent with the predictive coding framework, in which expectation suppression arises as a consequence of top-down fulfilled predictions. Here, we provided evidence for an alternative explanation for this phenomenon (Moors, 2015). We propose that the participants instead of predicting the inducers, might amodally complete them as occluded circles behind the illusory triangle. In turn, the amodally completed circles could induce larger adaptation effects compared with other non-illusory conditions in which amodal completion does not take place. This alternative interpretation highlights the need to carefully reevaluate how much empirical evidence we have gained in favor of predictive coding when testing perceptual effects such as the Kanizsa illusion.
